# Bioinformatics Analysis of the Interaction of miRNAs and piRNAs with Human mRNA Genes Having di- and Trinucleotide Repeats

**DOI:** 10.3390/genes13050800

**Published:** 2022-04-29

**Authors:** Ayaz Belkozhayev, Raigul Niyazova, Cornelia Wilson, Nurlan Jainakbayev, Anna Pyrkova, Yeldar Ashirbekov, Aigul Akimniyazova, Kamalidin Sharipov, Anatoliy Ivashchenko

**Affiliations:** 1 Department of Biotechnology, Al-Farabi Kazakh National University, Almaty 050040, Kazakhstan; ayaz_jarkent@mail.ru (A.B.); raiguln@mail.ru (R.N.); 2 M.A. Aitkhozhin Institute of Molecular Biology and Biochemistry, Almaty 050012, Kazakhstan; eldarasher@mail.ru (Y.A.); skamalidin@mail.ru (K.S.); 3 School of Human and Life Sciences, Canterbury Christ Church University, Sandwich CT13 9FF, UK; cornelia.wilson@canterbury.ac.uk; 4 Department of Molecular & Clinical Cancer Medicine, Institute of Translational Medicine, University of Liverpool, Liverpool L69 3BX, UK; 5 Department of Public Health, Kazakh-Russian Medical University, Almaty 050022, Kazakhstan; tnd1101@mail.ru; 6 Center for Bioinformatics and Nanomedicine, Almaty 050060, Kazakhstan; anna.pyrkova@kaznu.kz; 7 Higher School of Medicine, Faculty of Medicine and Healthcare, Al-Farabi Kazakh National University, Almaty 050040, Kazakhstan; akimniyazova.aigul@med-kaznu.com; 8 Department of Biochemistry, Asfendiyarov Kazakh National Medical University, Almaty 050000, Kazakhstan

**Keywords:** nucleotide repeats, gene, miRNA binding, piRNA, mRNA, neurodegenerative disorders, oncological diseases, diabetes

## Abstract

The variability of nucleotide repeats is considered one of the causes of diseases, but their biological function is not understood. In recent years, the interaction of miRNAs and piRNAs with the mRNAs of genes responsible for developing neurodegenerative and oncological diseases and diabetes have been actively studied. We explored candidate genes with nucleotide repeats to predict associations with miRNAs and piRNAs. The parameters of miRNAs and piRNA binding sites with mRNAs of human genes having nucleotide repeats were determined using the MirTarget program. This program defines the start of the initiation of miRNA and piRNA binding to mRNAs, the localization of miRNA and piRNA binding sites in the 5′-untranslated region (5′UTR), coding sequence (CDS) and 3′-untranslated region (3′UTR); the free energy of binding; and the schemes of nucleotide interactions of miRNAs and piRNAs with mRNAs. The characteristics of miRNAs and piRNA binding sites with mRNAs of 73 human genes were determined. The 5′UTR, 3′UTR and CDS of the mRNAs of genes are involved in the development of neurodegenerative, oncological and diabetes diseases with GU, AC dinucleotide and CCG, CAG, GCC, CGG, CGC trinucleotide repeats. The associations of miRNAs, piRNAs and candidate target genes could be recommended for developing methods for diagnosing diseases, including neurodegenerative diseases, oncological diseases and diabetes.

## 1. Introduction

Dinucleotide and trinucleotide repeats are quite common in genes [1]. They are located in the 5′UTR, CDS, or 3′UTR, and therefore their role is not only in the coding of the amino acids of proteins. The presence of nucleotide repeats in the 5′UTR, CDS, or 3′UTR can affect both the transcription rate and the translation process [2,3,4]. The best-known effect on translation is miRNA molecules, which can bind to mRNA and block protein synthesis [5,6,7]. In this case, one miRNA can regulate the translation of several mRNAs if they contain binding sites (BSs) for this miRNA [8,9,10,11,12,13]. The diversity of translation regulation is expressed both in the composition of nucleotides of miRNA binding sites and in a variety of variants of site arrangement in mRNA. The mRNA can contain several BSs of one miRNA located separately in different parts of the mRNA or a sequential overlap of nucleotides of the BSs of one miRNA [10]. Recently, an overlap of BS nucleotides of several miRNAs was revealed, and this region of mRNA was named the cluster of miRNA BSs [10]. As a result of this arrangement of BS miRNAs, competition for binding to the mRNA arises between these miRNAs. The miRNA BSs, consisting of different sets of dinucleotides and trinucleotides, confer both specificities for different miRNAs and the ability of miRNAs with similar nucleotide compositions to bind at the same site with different free interaction energies. In recent years, various diseases associated with changes in miRNA concentration have been identified, and there is a need to determine which miRNAs can regulate the expression of candidate genes for these diseases [14]. This need is primarily due to establishing unique target genes for miRNAs or competing miRNAs for the diagnosis and therapy of diseases.

**Figure 1:**
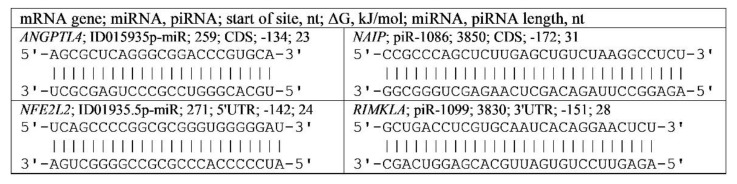
The characteristics of miRNA, piRNA, and mRNA (*ANGPTL4*, *NFE2L2*, *NAIP*, and *RIMKLA*) interactions with ΔG/ΔGm equal 100%.

More than ten years ago, piRNAs were identified [15,16,17], but their biological role has not been studied in detail [18]. Based on the similarity of several properties of miRNA and piRNA, we hypothesized that piRNAs could bind to mRNA and regulate translation. The similarity of miRNA and piRNA lies in the binding to the Argonaut and PIWI proteins [19,20], respectively, with the formation of complexes facilitating the delivery of miRNA and piRNA to mRNA and the formation of stable bonds with mRNA. Furthermore, we have identified the ability of piRNAs to bind to the mRNAs of many genes and have shown that piRNAs can bind more efficiently than miRNAs to the mRNAs of target genes (Figure 1). In this regard, the aim of this work was to establish the possibility of dinucleotide and trinucleotide repeats in the 5′UTR, CDS, and 3′UTR of mRNA binding miRNA and piRNA.

The analysis of the effect of miRNA on genes containing the trinucleotide repeats showed that such repeats contained in CDS mRNAs encode polyA, polyG, polyQ, and polyP oligopeptides. Several studies have shown that these oligopeptides are associated with Huntington’s diseases [21,22], spinocerebellar ataxias, dentatorubral-pallidoluysian atrophy, and spinal bulbar muscular atrophy diseases [23,24]. One of the causes of these diseases may be the binding of miRNAs or piRNAs to mRNAs containing nucleotide repeats. If these genes are candidate genes for various diseases, then this will lead to the corresponding diseases. Furthermore, if miRNA or piRNA regulates the expression of some genes, then there is a system of regulation of these genes with the help of a group of these miRNAs and piRNAs. These assumptions show the need to describe the interaction of miRNA and piRNA with mRNA genes containing dinucleotide and trinucleotide repeats and involved in the development of various diseases.

## 2. Materials and Methods 

The nucleotide sequences of the 17,494 mRNAs of targeted genes were downloaded from NCBI GenBank (http://www.ncbi.nlm.nih.gov accessed on 5 January 2020). The nucleotide sequences of the miRNAs were taken from miRBase v.22 (http://www.mirbase.org/accessed on 5 January 2020). Three million piRNAs are available in the piRBase database (http://pirbase.org/database/piRNA/, accessed on 18 November 2021). The miRNAs and piRNA BSs in the mRNAs of several genes were predicted using the MirTarget program [25]. This program defines the following features of miRNAs and piRNAs binding to mRNA: (a) the initiation of the miRNA binding to the mRNAs from the first nucleotide of the mRNAs; (b) the localization of the miRNAs, piRNA BSs in the 5′-untranslated region (5′UTR), coding domain sequence (CDS), and 3′-untranslated region (3′UTR) of the mRNAs; (c) the schemes of nucleotide interactions between miRNAs, piRNAs and mRNAs; and (d) the free energy of the interaction between miRNAs, piRNAs and mRNA (ΔG, kJ/mol); and the ratio ΔG/ΔGm (%) is determined for each site. The ΔGm equals the free energy of the miRNAs and piRNAs binding with their fully complementary nucleotide sequence. The MirTarget program finds hydrogen bonds between adenine (A) and uracil (U), guanine (G) and cytosine (C), G and U, and A and C. Regarding the free energy of interactions (ΔG), a pair of G and C is equal to 6.37 kJ/mole, a pair of A and U is equal to 4.25 kJ/mole, and a pair of G and U or A and C is equal to 2.12 kJ/mole. The distances between bound A and C (1.04 nm) and G and U (1.02 nm) are similar to those between bound G and C and between bound A and U and are equal to 1.03 nm [26,27]. The numbers of hydrogen bonds in the G–C, A–U, G–U, and A–C interactions were 3, 2, 1, and 1, respectively. By comparison, MirTarget differs from other programs [28,29] in terms of finding the BSs of miRNA on the mRNAs in the following: (1) it accounts for the interaction of the miRNAs, piRNAs with mRNA over the entire miRNAs, and piRNA sequence; (2) it considers noncanonical G–U and A–C pairs; and (3) it calculates the free energy of the interaction of the miRNAs and piRNAs with mRNA. When two or more miRNAs or piRNAs are bound with one mRNA, or if the BSs of two different miRNAs coincide in part, the preferred miRNA binding site is considered to be the one for which the free binding energy is greater. The adequacy of the program in terms of finding BSs has been confirmed in several publications [30,31,32,33]. The MirTarget program predicts the BSs of human miRNAs and piRNAs equally well (Figure 1). 

There are no “wet” experiments to find BSs for all miRNA or piRNA nucleotides with BSs and to determine the free energy of their interaction. In addition, widely used programs do not consider the interaction of noncanonical nucleotide pairs, which significantly distorts the value of the free energy of interaction between miRNAs, piRNAs and BSs [28,29]. A consideration of schemes shows which nucleotides of noncanonical pairs and which position decrease the maximum possible energy of interaction between miRNAs, piRNAs and BSs. The schemes can be verified manually by finding the predicted miRNA and piRNA BSs in the mRNA nucleotide sequence in the available databases. 

## 3. Results 

### 3.1. The BSs of miRNAs in mRNAs of Genes Having Dinucleotide Repeats Associated with Neurodegenerative Disorders

BSs of miR-466, ID00436.3p-miR, miR-574-5p and ID00470.5p-miR were identified in the 3′UTR mRNAs of 15 genes associated with neurodegenerative disorders, such as autism spectrum, Parkinson’s disease, schizophrenia, depressive disorders, Alzheimer’s, mental depression, amyotrophic lateral sclerosis, anxiety, and intellectual disability diseases (Table S1).

miR-466, ID00436.3p-miR, miR-574-5p and ID00470.5p-miR have BSs in the 3′UTR mRNA of the *ADCYAP1R1*, *BACH1*, *CACNG8*, *CD2AP*, *CD36*, *HPS4*, *PCK1*, *SAMD4A*, *SH3PXD2A*, *VAPB*, *AMOTL1*, *BTBD9*, *C10orf71*, *DPYSL5* and *FOXN3* genes. The associations of these genes with miR-466 and ID00436.3p-miR are characterized by the same values of the free interaction energy. Therefore, for the diagnosis of diseases, it is necessary to simultaneously measure the mRNA concentrations of ten target genes, miR-466 and ID00436.3p-miR. Otherwise, it is impossible to determine which association is responsible for the disease.

Similarly, it is necessary to simultaneously estimate the concentrations of the mRNA of the *AMOTL1*, *BTBD9*, *C10orf71*, *DPYSL5*, and *FOXN3* genes, miR-574-5p and ID00470.5p-miR. The mRNAs of genes with AC and GU dinucleotide repeats bind with miR-466, ID00436.3p-miR, miR-574-5p and ID00470.5p-miR with ΔG/ΔGm values ranging from 89% to 93% (Table S1). This small difference in ΔG/ΔGm values requires the determination of the concentrations of mRNA of candidate genes to identify the gene responsible to a greater extent for the development of the disease.

As shown in Table S1, the mRNA of the *ADCYAP1R1*, *BACH1*, *CACNG8*, *CD2AP*, *CD36*, *HPS4*, *PCK1*, *SAMD4A*, *SH3PXD2A* and *VAPB* genes, which are involved in neurodegenerative diseases with GU repeats, contains several BSs for miR-466 and ID00436.3p-miR. The number of these repeat BSs (clusters) varies from 4 to 10. This indicates an increase in the dependence of gene expression on these miRNAs. This also indicates the reliability of the interaction between the miRNAs and target genes. miR-466 and ID00436.3p-miR interact with the mRNA of these genes at the same free energy of −105 ± 1 kJ/mole. All of these miRNA BSs are located in the 3′UTR.

miR-574-5p and ID00470.5p-miR bind to the mRNA of the *AMOTL1*, *BTBD9*, *C10orf71*, *DPYSL5* and *FOXN3* genes with AC repeats, and BSs are located in the 3′UTR. The free energy values of these miRNA interactions with the target genes were equal to −111 ± 3 kJ/mole (Table S1). The number of BSs in the cluster in these miRNAs ranged from 7 to 13. This shows an increase in the dependence of target gene expression on miR-574-5p and ID00470.5p-miR.

**Figure 2:**
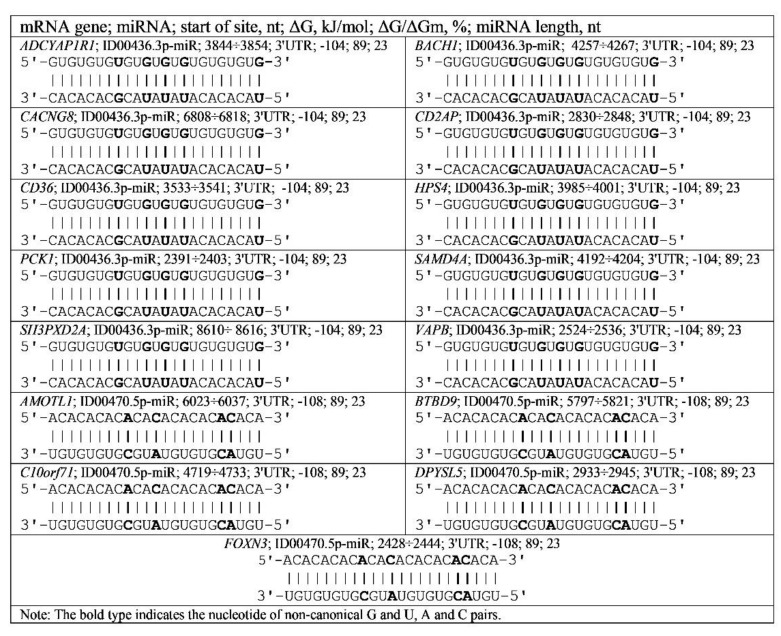
Schemes of ID00436.3p-miR and ID00470.5p-miR binding with mRNA of genes having GU and AC dinucleotide repeats in the 3′UTR.

Schemes show a total complementarity between canonic (A-U, G-C) and noncanonical (A-C, G-U) nucleotides of miRNAs and their BSs (Figure 2). BSs in mRNA *ADCYAP1R1*, *BACH1*, *CACNG8*, *CD2AP*, *CD36*, *HPS4*, *PCK1*, *SAMD4A*, *SH3PXD2A*, *VAPB*, *AMOTL1*, *BTBD9*, *C10orf71*, *DPYSL5* and *FOXN3* genes, indicating a role of noncanonical pairs in regulating the expression of their target genes. 

The quantitative characteristics of miRNA interactions with mRNA shown in the schemes (free energy interaction and the ratio of ΔG/ΔGm values) cannot be obtained in the so-called “wet” experiments. These characteristics are fundamental when considering the competition between miRNAs that bind to mRNAs. For example, ID00436.3p-miR and miR-466, as well as ID00470.5p-miR and miR-574-5p, compete for binding in the same cluster. miR-466 will bind to a greater extent than ID00436.3p-miR because the free energy of miR-466 binding is greater than ID00436.3p-miR. Similarly, miR-574-5p will bind more strongly to the mRNA of the target gene than ID00470.5p-miR because the free energy of interaction with mRNA is greater in miR-574-5p (Table S1). 

### 3.2. The BSs of miRNAs in mRNAs of Genes Having Dinucleotide Repeats Associated with Oncological Diseases

The miR-466, ID00436.3p-miR, miR-574-5p and ID00470.5p-miR, can bind with mRNAs of 18 genes having GU and AC dinucleotide repeats. The characteristics of BSs of miR-466 and ID00436.3p-miR in 3′UTR mRNA of six genes with ΔG/ΔGm values equal to 89–91% are indicated in Table S2. 

miR-574-5p and ID00470.5p-miR bind to the 3′UTR mRNA of 12 genes with ΔG/ΔGm values equal to 89–93%. Target genes participate in the development of oncological diseases, including adrenal cortical adenoma, neuroblastoma, malignant neoplasms, colorectal carcinoma, malignant neoplasm of the breast, malignant neoplasm of the skin, liver carcinoma, brain neoplasms, differentiated thyroid cancer, cancer stem cells and pancreatic cancer (Table S2). The *ABLIM1*, *BAZ2A*, *CBX3*, *CD3EAP*, *CDK6* and *REEP3* genes, which are involved in oncological diseases, contain BSs for miR-466 and ID00436.3p-miR with GU repeats in the 3′UTR with −105 ± 1 kJ/mole free energy. The number of repeating sites (clusters) varies from 4 to 14. miR-574-5p and ID00470.5p-miR bind to *ANO8*, *ARHGAP35*, *ARRB1*, *BDH1*, *DOK6*, *E2F8*, *EHD3*, *FAM163A*, *GLI2*, *MNT*, *WNT4* and *ZRANB1* mRNAs in regions containing AC repeats located in the 3′UTR. The free energy of the interaction of these miRNAs with the target genes was equal to −111 ± 3 kJ/mole. The number of BSs of these miRNAs varied from 4 to 11.

**Figure 3:**
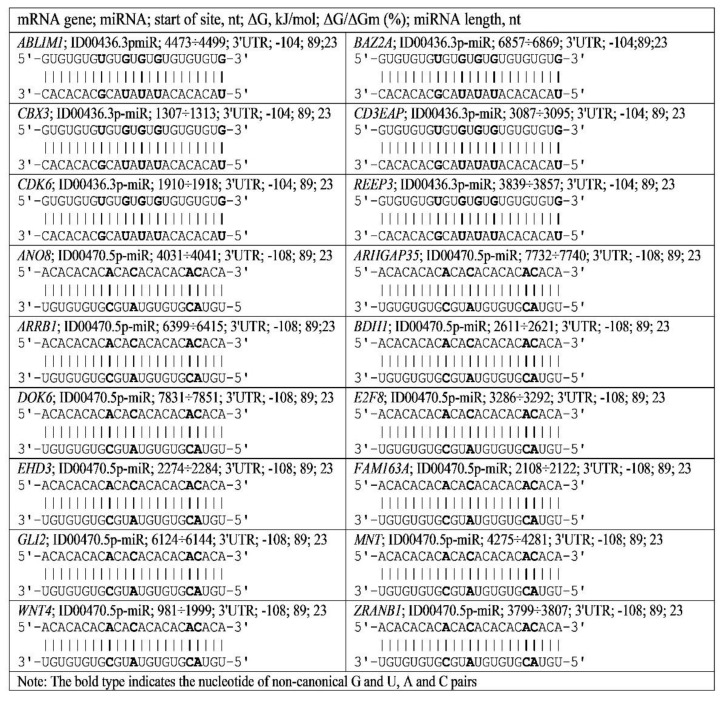
Schemes of ID00436.3p-miR and ID00470.5p-miR binding with mRNAs of genes having GU and AC nucleotide repeats.

The results shown in Figure 3 confirm the conclusions drawn from the results shown in Table S2. The obtained characteristics of the interaction of miRNA with mRNA of cancer candidate genes should also be taken into account when considering the interaction of miRNA with mRNA. For example, miR-466 binds with the *ABLIM1*, *BAZ2A*, *CBX3*, *CD3EAP*, *CDK6* and *REEP3* mRNAs to a greater extent than ID00436.3p-miR because the free energy binding of miR-466 is greater than that of ID00436.3p-miR (Table S2). Similarly, miR-574-5p will bind more strongly to the mRNA of the target genes *ANO8*, *ARHGAP35*, *ARRB1*, *BDH1*, *DOK6*, *E2F8*, *EHD3*, *FAM163A*, *GLI2*, *MNT*, *WNT4* and *ZRANB1* than ID00470.5p-miR because the free energy of interaction with mRNA is greater in miR-574-5p (Table S2). As the MirTarget program considers the interaction of the noncanonical pairs A-C and G-U, the interaction of miRNAs and mRNAs preserves the spiral structures of both molecules, and therefore, stacking interactions are found between all nucleotides of miRNA and mRNA, which stabilize the duplex [27]. 

### 3.3. The BSs of miRNAs in mRNAs of Genes Having Dinucleotide Repeats Associated with Diabetes

Table S3 shows BSs of miR-466, ID00436.3p-miR, miR-574-5p and ID00470.5p-miR in regions with GU and AC dinucleotide repeats in the 3′UTR mRNAs of the *BACH2*, *FASLG*, *HEMGN*, *IGF2R*, *ACVR2B*, *AFF3*, *CAMK2N1*, *EP300* and *FAM167A* genes involved in the development of diabetes. The free energy of the interaction of miR-466 and ID00436.3p-miR with mRNA of the *BACH2*, *FASLG*, *HEMGN* and *IGF2R* genes, which are involved in diabetes diseases, is −105 ± 1 kJ/mole with GU repeats in the 3′UTR. The number of these repeat sites (clusters) varied from 4 to 14 (Table S3). The binding sites for miR-574-5p and ID00470.5p-miR were identified in the 3′UTR of the *ACVR2B*, *AFF3*, *CAMK2N1*, *EP300* and *FAM167A* mRNAs with a free energy of −111 ± 3 kJ/mole with AC repeats. 

**Figure 4:**
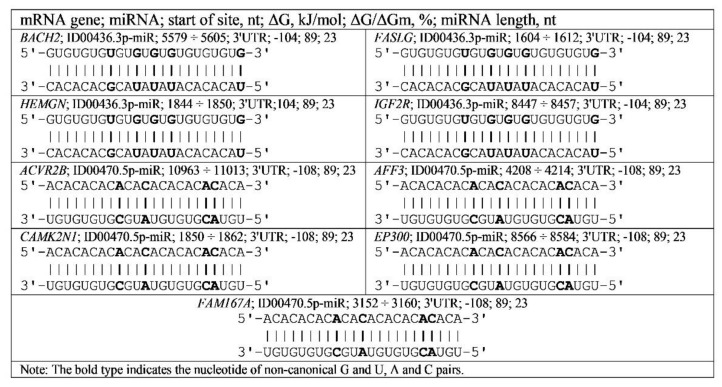
Schemes of ID00436.3p-miR and ID00470.5p-miR binding in mRNA genes having GU and AC nucleotide repeats in 3′UTR.

As a result, schemes of ID00436.3p-miR and ID00470.5p-miR binding with mRNA of the *BACH2*, *FASLG*, *HEMGN*, *IGF2R*, *ACVR2B*, *AFF3*, *CAMK2N1*, *EP300* and *FAM167A* genes with complete complementarity of BSs were revealed (Figure 4). The schemes show the formation of hydrogen bonds between all nucleotides of miRNAs and their BSs in mRNA. In the diagrams of Figure 4, the noncanonical pairs GU and AC clearly play an important role in increasing the free energies of the interaction between miRNA and mRNA and maintaining the double-helix structure of miRNA and mRNA by increasing the stacking interaction. Interestingly, mRNAs of the *BACH2*, *FASLG*, *HEMGN*, *IGF2R*, *ACVR2B*, *AFF3*, *CAMK2N1*, *EP300* and *FAM167A* genes contained multiple BSs for miR-466, ID00436.3p-miR, miR-574-5p and ID00470.5p-miR in the same regions, for example, 14 BSs of miR-466 and ID00436.3p-miR in *BACH2*; five BSs in *FASLG*; four BSs in *HEMGN*; five and six BSs in *IGF2R* with ΔG −104 kJ/mole to −106 kJ/mole. miR-574-5p and ID00470.5p-miR have 26 BSs in *ACVR2B*, four BSs in *AFF3*, seven BSs in *CAMK2N1*, ten BSs in *EP300* and five BSs in *FAM167A* with ΔG −108 kJ/mole to −113 kJ/mole.

### 3.4. Binding of miRNA and piRNA to mRNA of Genes with Trinucleotide Repeats

**Figure 5:**
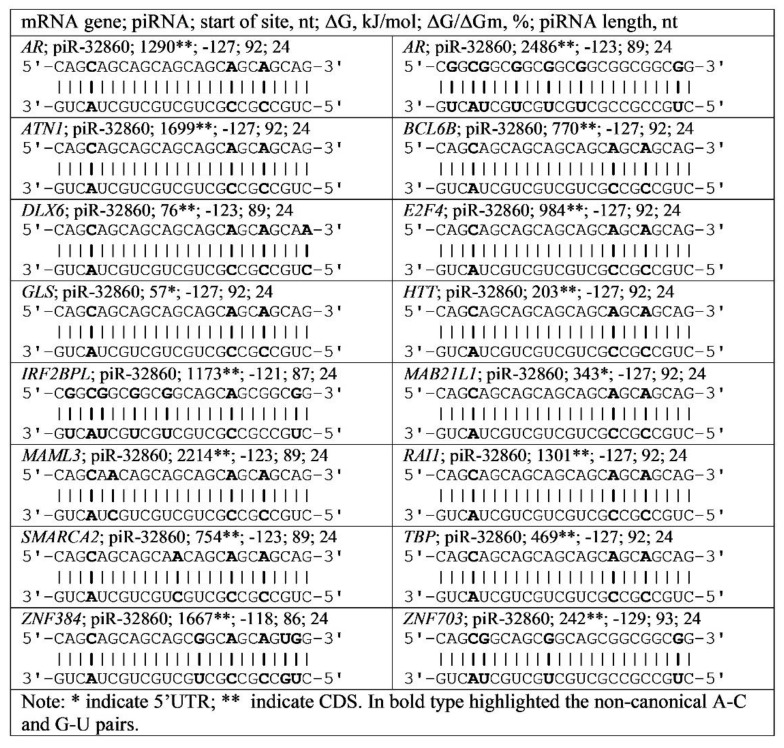
Schemes of piR-32860 interaction with mRNA regions of various genes containing CAG and CGG triplets.

Repetitive nucleotide triplets are found in many genes. We identified binding sites for miRNA and piRNA in the 5′UTR and CDS mRNA of these genes (Tables S4 and S5). The data presented show that some piRNAs bind to the mRNA of many genes by interacting with repeats of specific triplets. For example, piR-32860 interacts with CAG and CGG triplets located in the mRNA of *AR*, *ATN1*, *BCL6B*, *DLX6*, *E2F4*, *GLS*, *HTT*, *IRF2BPL*, *MAB21L1*, *MAML3*, *RAI1*, *SMARCA2*, *TBP*, *ZNF384*, and *ZNF703*. Both types of repeats of CAG and CGG triplets are localized in the *AR* gene mRNA (Figure 5). 

**Figure 6:**
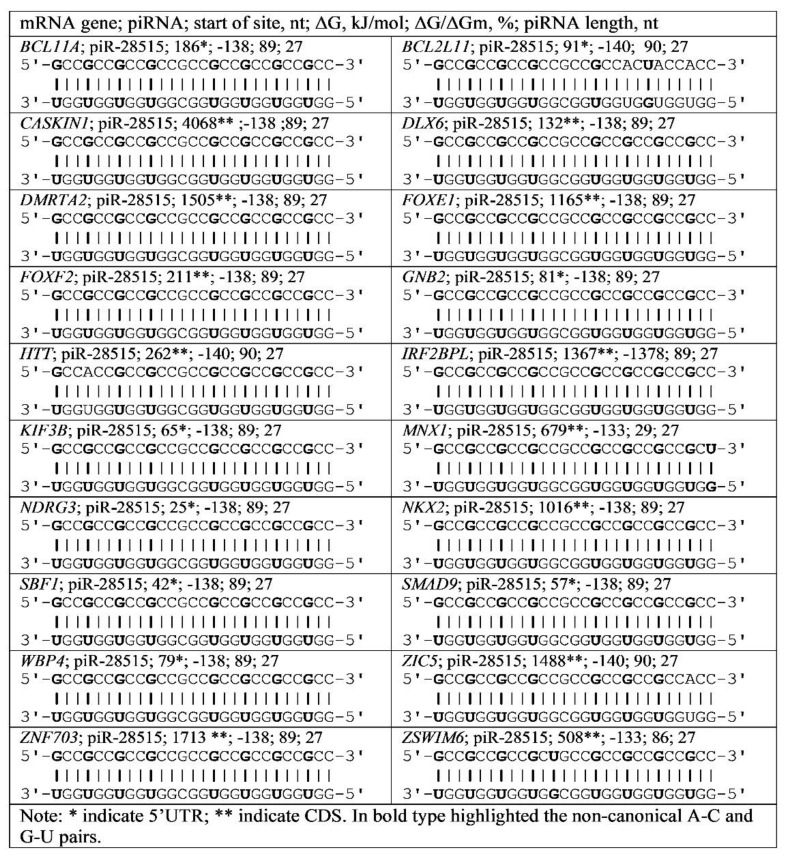
Schemes of piR-28515 interaction with mRNA regions of various genes containing GCC triplets.

Figure 6 shows the scheme of piR-28515 binding with repeats of the GCC triplet in the 5′UTR and CDS mRNA of the *BCL11A*, *BCL2L11*, *CASKIN1*, *DLX6*, *DMRTA2*, *FOXE1*, *FOXF2*, *GNB2*, *HTT*, *IRF2BPL*, *KIF3B*, *MNX1*, *NDRG3*, *NKX2*, *SBF1*, *SMAD9*, *WBP4*, *ZIC5*, *ZNF703* and *ZSWIM6* genes. 

BSs were also identified for piR-28385 in the 5′UTR and CDS mRNA of *BCL11A*, *BCL2L11*, *DLX6*, *DMRTA2*, *CASKIN1*, *FOXE1*, *FOXF2*, *GNB2*, *HTT*, *IRF2BPL*, *KIF3B*, *MNX1*, *NDRG3*, *NKB4*, *SMP9*, *SBF ZIC5*, *ZNF703*, and *ZSWIM6* containing GCC triplets. The free binding energy of piR-28385 was approximately −20 kJ/mole higher than that of piR-28515 (Tables S4 and S5). 

**Figure 7:**
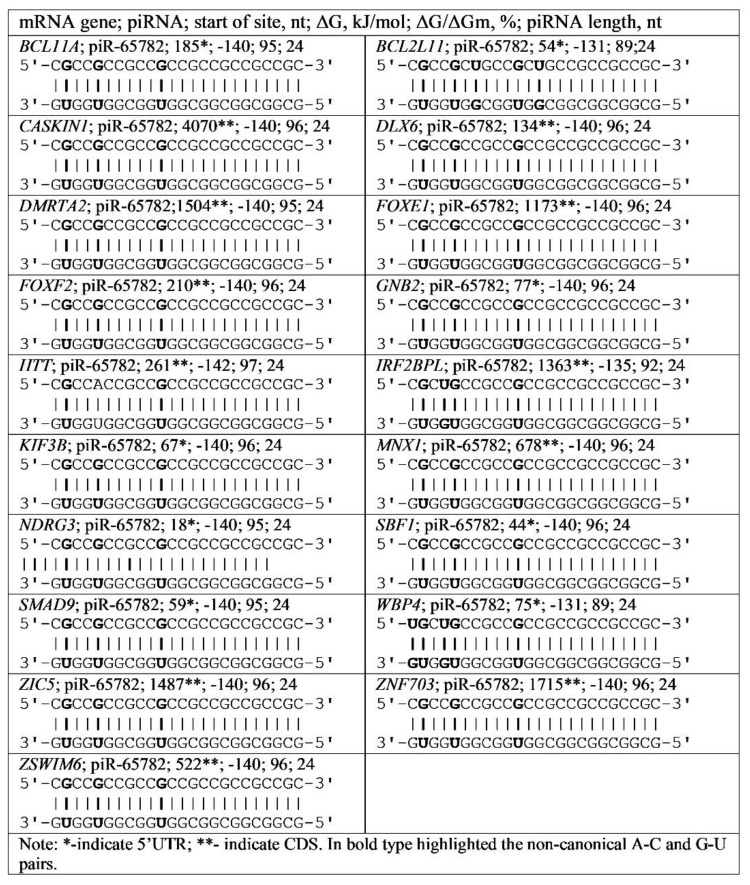
Schemes of piR-65782 interaction with mRNA regions of various genes containing CGC triplets.

piR-65782 and piR-478 bound the CGC triplet repeats in mRNA of the *BCL11A*, *BCL2L11*, *CASKIN1*, *DLX6*, *DMRTA2*, *FOXE1*, *FOXF2*, *GNB2*, *HTT*, *IRF2BPL*, *KIF3B*, *MNX1*, *NDRG3*, *SBF1*, *SMAD9*, *WBP4*, *ZIC5*, *ZNF703*, and *ZSWIM6* genes. Examples of interaction schemes for piR-65782 genes are shown in Figure 7. 

The quantitative characteristics of the interaction of miRNA and piRNA with the mRNA of genes containing various triplets indicate that these repeated triplets are located in the 5′UTR and CDS; that is, they can encode oligopeptides or not. Moreover, the coding of oligopeptides can occur in different reading frames. Consequently, the biological role of genes containing repeated nucleotide triplets is associated with the dependence of their expression on miRNAs and piRNAs. 

## 4. Discussion

Dinucleotide and trinucleotide repeats in genes have attracted the attention of researchers for a long time, but their functional purpose has not been sufficiently substantiated. The detection of such repeats in the 5′UTR, CDS and 3′UTR is interpreted as a kind of genetic marker of the gene that is preserved in the process of evolution [34,35,36]. The location of these repeats in the 5′UTR, CDS and 3′UTR also does not have a convincing justification. Dinucleotide and trinucleotide repeats are claimed to be associated with genetic diseases [37,38,39]. However, the specific mechanisms for the participation of these repeats in the proposed processes remain at the level of assumptions. We have clearly shown that all repeats found in the mRNAs of different genes are binding sites for miRNAs and piRNAs.

miRNAs are known to be regulators of the translation process regardless of their localization in the 5′UTR, CDS and 3′UTR [3,4,5,6,7]. Moreover, the location of these repeats in CDS is not unambiguously associated with encoded oligopeptides even in orthologous genes, which emphasizes their role as regulators of gene expression rather than encoding a specific oligopeptide [9,10,11,12]. The role of piRNAs as regulators of gene expression, similar to miRNAs, established in the present work for the first time confirms their main biological role. The results of this work show the role of miRNA and piRNA as regulators of gene expression at the translation stage by binding them in the region of di- and trinucleotide repeats. The involvement of these repeats in various diseases is explained by their presence in the candidate genes of various diseases, which is also shown in this manuscript (Tables S1–S3).

Our results show that miRNAs and piRNAs can regulate the expression of genes with nucleotide repeats associated with the development of neurodegenerative, oncological and diabetes diseases. The data given in Supplementary Tables S4 and S5 together with the data in Figure 5, Figure 6 and Figure 7 show that one miRNA or piRNA can interact with several genes. That is, this miRNA or piRNA will bind more to the mRNA of the gene that is transcribed faster. At a constant rate of miRNA synthesis, this will lead to a decrease in the binding of this miRNA to the mRNA of other genes, which will lead to an increase in the synthesis of the corresponding proteins. Thus, miRNAs maintain a balance in the activity of genes dependent on them. In addition, one gene is the target of several piRNAs, resulting in competition between them for binding in one cluster of BSs. As a result of the association of several piRNAs and several genes, they form peculiar complexes of mutual regulatory participants in a physiological process.

An important property for many miRNAs and piRNAs is their binding to some genes. Why do we need control of the expression of these genes from miRNA and piRNA? piRNAs are synthesized at the beginning of embryogenesis, and their synthesis is retained only in reproductive and stem cells [40,41]. Then, during cell differentiation, there is a decrease in piRNA synthesis and an increase in miRNA synthesis. Therefore, there is a weak dependence of expression on piRNAs and an increased dependence of expression on miRNAs for genes in differentiated cells. The obtained results indicate that dinucleotide repeats are located predominantly in the 3′UTRs of the studied genes and that a limited set of miRNAs binds to them. The target genes of these miRNAs include candidate genes for cancer, neurodegenerative diseases, and diabetes. The piRNA target genes are candidate genes for cancer, neurodegenerative diseases, and diabetes containing BSs only from trinucleotide repeats and located only in the 5′UTR and CDS. It follows from these data that di- and trinucleotide repeats are binding sites for specific miRNAs and piRNAs. The biological role of miRNAs and piRNAs lies in the interrelated regulation of protein-coding gene expression.

## 5. Conclusions

In this study, we demonstrated in silico prediction of miRNAs and piRNAs binding with human mRNA genes having di- and trinucleotide repeats associated with socially significant diseases, including neurodegenerative and oncological diseases and diabetes. The characteristics of BS of miRNAs and piRNAs with mRNAs of human genes were determined. The average free energy of miRNA and piRNA binding in the mRNA of genes was greater in the 5′UTR and CDS than in the 3′UTR, which suggested preferential binding of miRNA and piRNA to the 5′UTR and CDS of the studied genes. The *BCL11A*, *BCL2L11*, *GLS*, *GNB2*, *KIF3B*, *MAB21L1*, *NDRG3*, *SBF1*, *SMAD9* and *WBP4* genes were selected as candidate target genes for miRNAs and piRNAs with binding sites in the 5′UTR of mRNA. Additionally, for the candidate genes *AR*, *ATN1*, *CASKIN1*, *DLX6*, *DMRTA2*, *E2F4*, *FOXE1*, *FOXF2*, *HTT*, *IRF2BPL*, *MAML3*, *MNX1*, *NKX2*, *RAI1*, *SMARCA2*, *TBP*, *ZIC5*, *ZNF384*, *ZNF703* and *ZSWIM6*, miRNAs and piRNA binding sites are located in the CDS with trinucleotide repeats. Based on these results, the associations of miRNAs and piRNA candidate target genes are recommended for developing methods for diagnosing neurodegenerative diseases, oncological diseases and diabetes.

## Data Availability

Data are contained within the present article.

## Supplementary Materials

The following are available online at https://www.mdpi.com/article/10.3390/genes13050800/s1, Table S1: Characteristics of miRNA interactions with the 3′UTR mRNAs of genes having GU and AC dinucleotide repeats and associated with neurodegenerative diseases. Table S2: Characteristics of miRNAs binding with the 3′UTR mRNAs of genes having GU and AC dinucleotide repeats and associated with oncological diseases. Table S3: Characteristics of miRNAs binding with the 3′UTR mRNAs of genes having GU and AC dinucleotide repeats and associated with diabetes diseases. Table S4: Characteristics of piRNA and miRNA interactions with the 5′UTR mRNAs of genes having trinucleotide repeats and associated with diseases. Table S5: Characteristics of piRNA and miRNA interactions with CDS mRNAs of genes having trinucleotide repeats and associated with diseases.
