# Supplementary material for: Bioinformatics Analysis of the Interaction of miRNAs and piRNAs with Human mRNA Genes Having di- and Trinucleotide Repeats
Source: Genes (Basel). 2022 Apr 29;13(5):800. doi: 10.3390/genes13050800 (PMC9141802; doi:10.3390/genes13050800)
Supplement: Supplementary file 1 [file genes-13-00800-s001.zip › genes-1688242-supplementary.pdf]

genes

Supplementary Materials to

Article

Bioinformatics prognosis of the interaction of miRNAs, piRNAs with human mRNA genes having di- and trinucleotide repeats

Ayaz Belkozhayev [1,2], Raigul Niyazova [1], Cornelia Wilson [3,4], Nurlan Jainakbayev [5], Anna Pyrkova [6], Yeldar Ashirbekov [2], Aigul Akimniyazova [7], Kamalidin Sharipov [2,8] and Anatoliy Ivashchenko [6,*]

1 Department of Biotechnology, Al-Farabi Kazakh National University, Almaty 050040, Kazakhstan; ayaz_jarkent@mail.ru (A.B.); raiguln@mail.ru (R.N.)
2 M.A. Aitkhozhin Institute of Molecular Biology and Biochemistry, Almaty 050012, Kazakhstan; eldarasher@mail.ru (Y.A.); skamalidin@mail.ru (K.S.)
3 School of Human and Life Sciences, Canterbury Christ Church University, Sandwich CT13 9FF, UK; cornelia.wilson@canterbury.ac.uk
4 Department of Molecular & Clinical Cancer Medicine, Institute of Translational Medicine, University of Liverpool, Liverpool L69 3BX, UK
5 Department of Public Health, Kazakh-Russian Medical University, Almaty 050022, Kazakhstan; tnd1101@mail.ru
6 Center for Bioinformatics and Nanomedicine, Almaty 050060, Kazakhstan; anna.pyrkova@kaznu.kz
7 Higher School of Medicine, Faculty of Medicine and Healthcare, Al-Farabi Kazakh National University, Almaty 050040, Kazakhstan; akimniyazova.aigul@med-kaznu.com
8 Department of Biochemistry, Asfendiyarov Kazakh National Medical University, Almaty 050000, Kazakhstan
* Correspondence: a.iavashchenko@gmail.com

Table S1. Characteristics miRNA interaction with 3′UTR mRNAs of genes having GU and AC dinucleotide repeats and associated with neurodegenerative diseases

Gene	miRNA	Start of BS, nt	ΔG, kJ/mol	ΔG/ΔGm, %	nt repeat	Disease [PMID]
ADCYAP1R1	miR-466<br>ID00436.3p-miR	3844÷3854 (6)<br>3844÷3854 (6)	-106<br>-104	91<br>89	GU	Autism spectrum disorders [30556326]; Parkinson disease [29230633]; Schizophrenia [28125634]; Depressive disorders [23280952]
BACH1	miR-466<br>ID00436.3p-miR	4257÷4267 (6)<br>4257÷4267 (6)	-106<br>-104	91<br>89	GU	Alzheimer's disease [15068237]
CACNG8	miR-466<br>ID00436.3p-miR	6808÷6818 (6)<br>6808÷6818 (6)	-106<br>-104	91<br>89	GU	Schizophrenia [27102562]
CD2AP	miR-466<br>ID00436.3p-miR	2830÷2848 (10<br>2830÷2848 (10)	-106<br>-104	91<br>89	GU	Mental depression [28630404];<br>Schizophrenia,<br>Alzheimer’s disease [30126816]
CD36	miR-466<br>ID00436.3p-miR	3533÷3539 (4)<br>3533÷3541 (5)	-106<br>-104	91<br>89	GU	Schizophrenia [22765916]
HPS4	miR-466<br>ID00436.3p-miR	3985÷3999 (8)<br>3985÷4001 (9)	-106<br>-104	91<br>89	GU	Schizophrenia [24168225]
PCK1	miR-466<br>ID00436.3p-miR	2391÷2401 (6)<br>2391÷2403 (7)	-106<br>-104	91<br>89	GU	Schizophrenia [18460190]
SAMD4A	miR-466<br>ID00436.3p-miR	4192÷4202 (6)<br>4192÷4204 (7)	-106<br>-104	91<br>89	GU	Schizophrenia [27846195]
SH3PXD2A	miR-466<br>ID00436.3p-miR	8610÷8616 (4)<br>8610÷8616 (4)	-106<br>-104	91<br>89	GU	Schizophrenia [24936775]
VAPB	miR-466<br>ID00436.3p-miR	2524÷2536 (7)<br>2524÷2536 (7)	-106<br>-104	91<br>89	GU	Amyotrophic lateral sclerosis [29628370];<br>Schizophrenia [19165527]
AMOTL1	miR-574-5p<br>ID00470.5p-miR	6023÷6037 (8)<br>6023÷6037 (8)	-113<br>-108	93<br>89	AC	Schizophrenia [22440650];<br>Alzheimer's disease [27075728]
BTBD9	miR-574-5p<br>ID00470.5p-miR	5797÷5821 (13)<br>5797÷5821 (13)	-113<br>-108	93<br>89	AC	Schizophrenia [23361623]
C10orf71	miR-574-5p<br>ID00470.5p-miR	4719÷4733 (8)<br>4719÷4733 (8)	-113<br>-108	93<br>89	AC	Alzheimer's disease [26830138]

DPYSL5	miR-574-5p<br>ID00470.5p-miR	2933÷2945 (7)<br>2933÷2945 (7)	-113<br>-108	93<br>89	AC	Alzheimer's disease [31233825]; Neurodevelopmental disorders [31446906];
FOXN3	miR-574-5p<br>ID00470.5p-miR	2428÷2444 (9)<br>2428÷2444 (9)	-113<br>-108	93<br>89	AC	Schizophrenia [21926974]

Note: In parentheses indicate the number of miRNAs BSs

Table S2. Characteristics of miRNAs binding with 3′UTR mRNAs of genes having GU and AC dinucleotide repeats and associated with oncological diseases.

Gene	miRNA	Start of BS, nt	ΔG, kJ/mole	ΔG/ΔGm, %	nt repeat	Disease [PMID]
ABLIM1	miR-466<br>ID00436.3p-miR	4473÷4499 (14)<br>4473÷4499 (14)	-106<br>-104	91<br>89	GU	Adrenal cortical adenoma [19218281]
BAZ2A	miR-466<br>ID00436.3p-miR	6857÷6869 (7)<br>6857÷6869 (7)	-106<br>-104	91<br>89	GU	Neuroblastoma [30064522]
CBX3	miR-466<br>ID00436.3p-miR	1307÷1313 (4)<br>1307÷1313 (4)	-106<br>-104	91<br>89	GU	Malignant neoplasms [30942427]
CD3EAP	miR-466<br>ID00436.3p-miR	3087÷3093 (4)<br>3087÷3095 (6)	-106<br>-104	91<br>89	GU	Malignant neoplasms [23624123]
CDK6	miR-466<br>ID00436.3p-miR	1910÷1918 (5)<br>1910÷1918 (5)	-106<br>-104	91<br>89	GU	Neuroblastoma [29073265]
REEP3	miR-466<br>ID00436.3p-miR	3839÷3857 (10)<br>3839÷3857 (10)	-106<br>-104	91<br>89	GU	Neuroblastoma [29042551]
ANO8	miR-574-5p<br>ID00470.5p-miR	4031÷4041 (6)<br>4031÷4041 (6)	-113<br>-108	93<br>89	AC	Malignant neoplasm of breast [20852631]
ARHGAP35	miR-574-5p<br>ID00470.5p-miR	7732÷7740 (5)<br>7732÷7740 (5)	-113<br>-108	93<br>89	AC	Malignant neoplasms [29934311]; Colorectal carcinoma [31332286]
ARRB1	miR-574-5p<br>ID00470.5p-miR	6399÷6415 (9)<br>6399÷6415 (9)	-113<br>-108	93<br>89	AC	Neoplasms [28442265]; Malignant neoplasm of breast [21497294]
BDH1	miR-574-5p<br>ID00470.5p-miR	2611÷2621 (6)<br>2611÷2621 (6)	-113<br>-108	93<br>89	AC	Malignant neoplasm of breast [23082721]; Malignant neoplasms [28025230]
DOK6	miR-574-5p<br>ID00470.5p-miR	7831÷7851 (11)<br>7831÷7851 (11)	-113<br>-108	93<br>89	AC	Malignant neoplasms [31486300]
E2F8	miR-574-5p<br>ID00470.5p-miR	3286÷3292 (4)<br>3286÷3292 (4)	-113<br>-108	93<br>89	AC	Malignant neoplasm of skin [27452520]; Liver carcinoma [28284560]
EHD3	miR-574-5p<br>ID00470.5p-miR	2274÷2284 (6)<br>2274÷2284 (6)	-113<br>-108	93<br>89	AC	Brain neoplasms [24306026]; Malignant neoplasms [31646426]; Liver carcinoma [28284560]
FAM163A	miR-574-5p<br>ID00470.5p-miR	2108÷2122 (8)<br>2108÷2122 (8)	-113<br>-108	93<br>89	AC	Malignant neoplasms [19671756]; Neoplasms, neuroblastoma [31496741]
GLI2	miR-574-5p<br>ID00470.5p-miR	6124÷6144 (11)<br>6124÷6144 (11)	-113<br>-108	93<br>89	AC	Malignant neoplasm of breast [31535669]
MNT	miR-574-5p<br>ID00470.5p-miR	4275÷4281 (4)<br>4275÷4281 (4)	-113<br>-108	93<br>89	AC	Malignant neoplasm of breast [16928695]; Malignant neoplasms [17577784]
WNT4	miR-574-5p<br>ID00470.5p-miR	1981÷1999 (10)<br>1981÷1999 (10)	-113<br>-108	93<br>89	AC	Malignant neoplasm of breast [30194396]; Malignant Neoplasms, carcinoma [29429512]
ZRANB1	miR-574-5p<br>ID00470.5p-miR	3799÷3807 (5)<br>3799÷3807 (5)	-113<br>-108	93<br>89	AC	Malignant neoplasm of breast [29669287]; Liver carcinoma [30686098]

Table S3. Characteristics of miRNAs binding with 3′UTR mRNAs of genes having GU and AC dinucleotide repeats and associated with diabetes diseases

Gene	miRNA	Start of BS, nt	ΔG, kJ/mol	ΔG/ΔGm, %	nt repeat	Disease [PMID]
BACH2	miR-466<br>ID00436.3p-miR	5579÷5605 (14)<br>5579÷5605 (14)	-106<br>-104	91<br>89	GU	Diabetes [30970177]; Diabetes mellitus, Insulin-dependent [24608439]
FASLG	miR-466<br>ID00436.3p-miR	1604÷1612 (5)<br>1604÷1612 (5)	-106<br>-104	91<br>89	GU	Diabetes mellitus, experimental [19820199]; Diabetes mellitus, insulin-dependent [17324464]
HEMGN	miR-466<br>ID00436.3p-miR	1844÷1850 (4)<br>1844÷1850 (4)	-106<br>-104	91<br>89	GU	Diabetes [28337877]; Diabetic Retinopathy [31275351]
IGF2R	miR-466<br>ID00436.3p-miR	8447÷8455 (5)<br>8447÷8457 (6)	-106<br>-104	91<br>89	GU	Diabetes [21852217]; Diabetes mellitus, non-insulin- dependent [18602895]
ACVR2B	miR-574-5p<br>ID00470.5p-miR	10963÷11013 (26)<br>10963÷11013 (26)	-113<br>-108	93<br>89	AC	Diabetes mellitus, non-insulin-dependent [11334431]
AFF3	miR-574-5p<br>ID00470.5p-miR	4208÷4214 (4)<br>4208÷4214 (4)	-113<br>-108	93<br>89	AC	Diabetes mellitus, insulin dependent [25751624]<br>Diabetic retinopathy [28779365]
CAMK2N1	miR-574-5p<br>ID00470.5p-miR	1850÷1862 (7)<br>1850÷1862 (7)	-113<br>-108	93<br>89	AC	Diabetes mellitus, non-insulin-dependent [31327268]
EP300	miR-574-5p<br>ID00470.5p-miR	8566÷8584 (10)<br>8566÷8584 (10)	-113<br>-108	93<br>89	AC	Diabetes mellitus [28886276]; [22228707]; Diabetic nephropathy [28444663]
FAM167A	miR-574-5p<br>ID00470.5p-miR	3152÷3160 (5)<br>3152÷3160 (5)	-113<br>-108	93<br>89	AC	Diabetes mellitus, insulin dependent [21765104]

Table S4. Characteristics piRNA and miRNA interaction with 5′UTR mRNAs of genes having trinucleotide repeats and associated with diseases

Genes	piRNA, miRNA	5′UTR positions, nt	Energy, kJ/mol	Score, %	piRNA, miRNA length, nt
BCL11A	piR-33638	177-189	-161	90	29
	piR-65782	179-197	-140	96	24
	ID00296.3p-miR	176-191	-142	91	25
	ID01702.3p-miR	179-194	-140	93	24
	piR-28385	180-192	-151	93	26
	piR-478	185-191	-153	92	28
	ID01895.5p-miR	181-187	-136	91	24
	ID02653.3p-miR	181-190	-113	85	22
	ID00756.3p-miR	182-188	-127	92	23
	miR-3960	181-193	-115	92	20
BCL2L11	piR-44682	54-93	-146	90	26
	piR-71189	67-79	-142	83	28
	piR-28514	67-91	-142	84	30
	ID00296.3p-miR	54-90	-134	85	25
	ID01702.3p-miR	54-90	-127	85	24
	miR-3960	56-92	-106	85	20
GLS	piR-16206	51-75	-121	85	26
	piR-hsa-70378	54-87	-140	87	28
	ID00372.5p-miR	53-77	-113	83	24
	miR-1322	54-87	-89	87	19
GNB2	piR-33638	72-93	-166	93	29
	piR-28812	74-95	-140	86	27
	piR-44682	74-98	-142	87	26
	piR-35158	75-87	-146	88	27
	piR-28513	78-90	-149	86	30
	ID00296.3p-miR	68-98	-136	86	25
	ID01804.3p-miR	74-101	-134	91	23
	ID01641.3p-miR	74-98	-132	89	24
	ID01702.3p-miR	74-98	-136	90	24
	novel-miR-1983-5p	78-93	-108	82	21
KIF3B	piR-33638	44-68	-151	85	29
	piR-28812	46-70	-144	88	27
	piR-28513	47-65	-144	84	30
	piR-44682	49-73	-149	91	26
	piR-57937	54-63	-155	87	30
	ID01804.3p-miR	49-70	-127	87	23
	ID00296.3p-miR	49-73	-149	95	25
	ID01641.3p-miR	49-73	-138	93	24
	ID01702.3p-miR	49-76	-129	86	24
	miR-3960	48-75	-113	90	20
	novel-miR-1011-3p	55-73	-110	90	20
MAB21L1	piR-16206	337-373	-119	84	26

	piR-32860	337-379	-115	83	24
	ID00372.5p-miR	342-372	-113	83	24
	miR-1322	343-379	-89	87	19
NDRG3	piR-478	18-36	-138	83	28
	piR-28812	15-39	-142	87	27
	piR-28513	16-31	-142	83	30
	piR-35158	16-31	-144	87	27
	piR-71189	19-31	-142	83	28
	piR-57937	20-29	-149	83	30
	ID00756.3p-miR	15-39	-117	85	23
	ID01804.3p-miR	15-45	-123	84	23
	ID02294.5p-miR	17-38	-125	86	24
	ID01041.5p-miR	18-36	-123	84	24
	ID02064.5p-miR	19-40	-123	85	23
	ID01895.5p-miR	20-35	-123	83	24
	novel-miR-1983-5p	19-40	-108	82	21
SBF1	piR-33638	36-46	-155	87	29
	piR-28812	38-44	-136	83	27
	piR-44682	38-47	-149	91	26
	piR-28385	42-48	-151	93	26
	ID01041.5p-miR	38-47	-125	86	24
	ID01804.3p-miR	38-50	-140	96	23
	ID00756.3p-miR	41-47	-125	91	23
	ID02064.5p-miR	42-54	-123	85	23
	ID02256.3p-miR	42-48	-115	83	22
	ID00722.5p-miR	43-52	-106	88	20
	miR-3960	43-52	-115	92	20
SMAD9	piR-33638	51-69	-146	82	29
	piR-28385	51-72	-134	83	26
	piR-28812	56-65	-151	92	27
	piR-69429	57-69	-144	89	26
	ID03229.5p-miR	51-69	-125	94	22
	ID02294.5p-miR	52-64	-121	83	24
	ID00296.3p-miR	56-65	-136	86	25
	ID00756.3p-miR	56-65	-125	91	23
	ID02296.5p-miR	57-66	-102	83	20
	ID00061.3p-miR	58-67	-117	85	22
	miR-3960	58-70	-115	92	20
	novel-miR-1983-5p	57-66	-108	82	21
WBP4	piR-33638	67-91	-149	83	29
	piR-44682	69-96	-140	86	26
	piR-28385	70-97	-138	86	26
	piR-28812	72-90	-136	83	27
	piR-28513	76-82	-144	84	30
	piR-478	81-87	-138	83	28
	ID01662.3p-miR	72-87	-123	87	23

ID00756.3p-miR	72-90	-115	83	23
ID00296.3p-miR	72-96	-129	82	25
ID01804.3p-miR	72-96	-125	86	23
miR-3960	74-95	-106	85	20
novel-miR-1983-5p	76-91	-108	82	21

Table S5. Characteristics piRNA and miRNA interaction with CDS mRNAs of genes having trinucleotide repeats and associated with diseases

Genes	piRNA, miRNA	CDS positions, nt	Energy, kJ/mol	Score, %	piRNA, miRNA length, nt
AR	piR-32860	1281-1335	-127	92	24
	piR-70378	1281-1326	-140	86	28
	ID00372.5p-miR	1283-1331	-115	84	24
	piR-37021	2476-2512	-134	89	25
	piR-32860	2477-2513	-123	89	24
	novel-miR-1639-3p	2483-2516	-106	85	21
ATN1	piR-32860	1687-1726	-127	92	24
	piR-70378	1693-1720	-140	86	28
	ID00372.5p-miR	1701-1722	-113	83	24
CASKIN1	piR-33638	4059-4074	-149	83	29
	piR-28812	4061-4073	-140	86	27
	piR-74279	4065-4071	-142	89	26
	piR-28385	4065-4077	-151	93	26
	piR-28513	4065-4080	-149	86	30
	ID00296.3p-miR	4061-4076	-129	82	25
	ID01804.3p-miR	4061-4076	-129	88	23
	ID00457.3p-miR	4063-4078	-127	94	22
	ID01641.3p-miR	4064-4076	-125	84	24
	ID01702.3p-miR	4064-4079	-129	86	24
	miR-3960	4066-4078	-115	92	20
	novel-miR-1983-5p	4065-4077	-108	82	21
DLX6	piR-33638	126-138	-151	85	29
	piR-28385	126-144	-140	87	26
	piR-28513	129-135	-144	84	30
	ID01041.5p-miR	125-137	-125	86	24
	ID01804.3p-miR	128-143	-125	86	23
	ID00296.3p-miR	131-140	-136	86	25
	ID01702.3p-miR	131-146	-129	86	24
	miR-3960	130-145	-108	86	20
	novel-miR-1983-5p	129-141	-108	82	21
DMRTA2	piR-33638	1493-1505	-157	88	29
	piR-46279	1499-1508	-136	89	25
	ID01702.3p-miR	1495-1510	-125	83	24
	ID01041.5p-miR	1501-1507	-125	86	24
	ID01641.3p-miR	1501-1510	-125	84	24
	miR-3960	1500-1512	-104	83	20
E2F4	piR-70378	981-1008	-140	87	28
	ID00372.5p-miR	983-998	-113	83	24
	miR-1322	981-1008	-89	87	19
FOXE1	piR-478	1153-1174	-138	83	28

	piR-33638	1155-1176	-146	82	29
	ID02064.5p-miR	1165-1183	-123	85	23
	novel-miR-1983-5p	1162-1177	-113	85	21
FOXF2	piR-33638	199-211	-149	83	29
	piR-28385	205-214	-136	84	26
	ID01641.3p-miR	201-216	-129	87	24
	ID00296.3p-miR	201-216	-140	89	25
	ID02256.3p-miR	208-214	-121	88	22
	novel-miR-1983-5p	211-220	-108	82	21
HTT	piR-44682	249-345	-136	83	26
	piR-33638	253-265	-146	82	29
	piR-28812	255-267	-136	83	27
	piR-478	261-267	-140	85	28
	ID01804.3p-miR	255-270	-121	83	23
	ID01702.3p-miR	258-270	-125	83	24
	ID01641.3p-miR	258-348	-125	84	24
	miR-3960	260-272	-110	88	20
IRF2BPL	piR-33638	1355-1373	-146	82	29
	piR-47476	1358-1367	-140	85	27
	piR-28812	1360-1372	-136	83	27
	ID01041.5p-miR	1360-1372	-125	86	24
	ID01804.3p-miR	1360-1380	-125	86	23
	ID02294.5p-miR	1362-1374	-127	87	24
	ID00296.3p-miR	1363-1375	-138	88	25
	ID01641.3p-miR	1363-1375	-127	86	24
	ID01702.3p-miR	1363-1378	-132	87	24
	miR-3960	1362-1380	-104	83	20
	novel-miR-1639-3p	1170-1276	-108	86	21
MAML3	piR-32860	2208-3045	-119	86	24
	piR-70378	2214-2253	-136	84	28
	ID00372.5p-miR	2222-2225	-113	83	24
	miR-1322	2214-3048	-89	87	19
MNX1	piR-33638	661-679	-149	83	29
	piR-44682	663-684	-146	90	26
	piR-28812	666-678	-136	83	27
	piR-28513	670-676	-144	84	30
	ID01804.3p-miR	663-678	-125	86	23
	ID01041.5p-miR	666-678	-125	86	24
	ID01702.3p-miR	669-684	-125	83	24
	miR-3960	668-686	-104	83	20
	novel-miR-1983-5p	673-685	-108	82	21
NKX2	piR-33638	1004-1022	-159	89	29
	piR-28812	1006-1021	-136	83	27
	piR-44682	1009-1027	-142	87	26
	piR-28513	1010-1016	-142	83	30
	piR-28385	1010-1025	-144	89	26

	ID00296.3p-miR	1006-1024	-132	84	25
	ID01804.3p-miR	1006-1027	-127	87	23
	ID01895.5p-miR	1014-1020	-129	87	24
	ID00061.3p-miR	1017-1026	-117	85	22
	miR-3960	1011-1026	-108	86	20
	novel-miR-1983-5p	1013-1025	-110	84	21
RAI1	piR-32860	1301-1319	-127	92	24
	miR-1322	1301-1312	-89	87	19
	ID00372.5p-miR	1303-1318	-113	83	24
SMARCA2	piR-70378	754-808	-136	84	28
	ID03311.5p-miR	771-786	-100	85	21
	ID00372.5p-miR	777-783	-113	83	24
	miR-1322	745-808	-85	83	19
TBP	piR-70378	466-538	-136	84	28
	ID03311.5p-miR	468-540	-100	85	21
	ID00372.5p-miR	504-537	-113	83	24
	miR-1322	502-544	-89	87	19
ZIC5	piR-41532	711-747	-140	84	31
	piR-28511	735-741	-132	86	27
	piR-26901	737-743	-134	86	27
	piR-32860	789-798	-117	85	24
ZIC5	piR-44682	1313-1496	-142	87	26
	piR-33638	1367-1494	-155	87	29
	piR-478	1481-1499	-138	83	28
	piR-28812	1472-1490	-146	90	27
	piR-28513	1473-1494	-144	84	30
	piR-28385	1473-1500	-146	91	26
	piR-57937	1474-1486	-151	85	30
	piR-28514	1476-1491	-142	84	30
	ID01804.3p-miR	1313-1325	-121	83	23
	ID01641.3p-miR	1313-1496	-132	89	24
	ID00296.3p-miR	1313-1496	-142	91	25
	D00457.3p-miR	1471-1498	-121	89	22
	ID02294.5p-miR	1471-1592	-121	83	24
	ID01041.5p-miR	1472-1487	-125	86	24
	ID00756.3p-miR	1472-1493	-121	88	23
	ID01702.3p-miR	1472-1496	-125	83	24
	ID01804.3p-miR	1472-1499	-127	87	23
	novel-miR-1983-5p	1473-1491	-108	82	21
ZNF384	piR-32860	1661-1798	-115	83	24
	piR-16206	1771-1789	-119	84	26
	ID00372.5p-miR	1773-1791	-113	83	24
	miR-1322	1771-1801	-89	87	19
ZNF703	piR-478	1712-1724	-138	83	28
	piR-33638	1707-1716	-159	89	29
	piR-28385	1707-1719	-140	87	26

	ID01041.5p-miR	1706-1715	-125	86	24
	ID01804.3p-miR	1706-1721	-123	84	23
	ID00296.3p-miR	1709-1718	-136	86	25
	ID01702.3p-miR	1709-1721	-129	86	24
	novel-miR-1011-3p	1712-1718	-104	84	20
ZSWIM6	piR-33638	499-526	-157	88	29
	piR-28812	501-525	-138	84	27
	piR-44682	501-534	-151	92	26
	piR-28513	505-523	-144	84	30
	piR-69429	505-532	-140	87	26
	piR-65782	495-534	-122	84	24
	ID00296.3p-miR	501-528	-134	85	25
	ID01702.3p-miR	501-531	-127	85	24
	ID01804.3p-miR	501-531	-129	88	23
	ID02294.5p-miR	503-527	-121	83	24
	miR-3960	500-533	-106	85	20
	novel-miR-1011-3p	522-531	-104	84	20
